# Aptamer-functionalized Hybrid Nanoparticles to Enhance the Delivery of Doxorubicin into Breast Cancer Cells by Silencing P-glycoprotein

**DOI:** 10.29245/2578-2967/2020/1.1176

**Published:** 2020

**Authors:** Sruti Chandra, Hoang Michael Nguyen, Kylar Wiltz, Nicholas Hall, Shanzay Chaudhry, George Olverson, Tarun Mandal, Srikanta Dash, Anup Kundu

**Affiliations:** 1 Department of Biology, Xavier University of Louisiana, New Orleans, Louisiana; 2 Center for Nanomedicine and Drug Delivery, Xavier University College of Pharmacy, New Orleans, Louisiana; 3 Department of Pathology and Laboratory Medicine, Tulane University Health Sciences Center, New Orleans, Louisiana

**Keywords:** siRNA, P-glycoprotein, Nanoparticle, Aptamer, Doxorubicin, Breast Cancer, Drug Resistance

## Abstract

**Objective:**

The MDR of metastatic breast cancer cells is accompanied by the overexpression of P-gp transporter. This study has been focused to determine whether silencing the expression of P-gp by aptamer-labeled siRNA nanoparticles could enhance the delivery of doxorubicin into breast cancer cells in culture.

**Methodology:**

The nanoparticle F-31 was prepared using DOTAP, cholesterol, and PLGA, and then incorporating Mal-PEG to facilitate aptamer-binding. The nanoparticles were surface-functionalized with aptamer A6, which targets Her-2 receptors overexpressed on the surface of breast cancer cells.

**Results:**

This study has shown that the uptake of Dox by Dox-resistant 4T1-R is significantly less than Dox-sensitive 4T1-S which is partly attributed to the higher expression of drug-efflux pump P-gp on the surface of the resistant cells. The targeted knockdown of P-gp has been enhanced when the particles carrying P-gp siRNA was labeled with aptamer. Concurrently, the uptake of Dox into the Dox-resistant 4T1-R breast cancer cells has increased significantly when the P-gp was silenced by P-gp siRNA-encapsulated aptamer-labeled nanoparticles.

**Conclusions:**

This preliminary study concludes that downregulating P-gp expression by targeted delivery of P-gp siRNA using aptamer-labeled lipid-based hybrid nanoparticles could effectively increase the intracellular trafficking of doxorubicin in Dox-resistant mouse breast cancer cells.

## Introduction

Although breast cancer incidences have declined in the recent past (by 37% from 1990–2013)^1,2^, it is still the second leading cause of death in women. According to cancer statistics^3^, 231,840 new breast cancer cases of women have registered in 2015 in the United States alone out of which 40,290 patients succumbed to death. Treatment options available at present include surgical excision, adjuvant radiation therapy, adjuvant chemotherapy and hormonal therapy. Chemotherapy involves the application of small molecule drugs like alkylating agents, anti-metabolites, anthracyclines and topoisomerase inhibitors. Over prolong exposure to the chemotherapeutic drug, cancer cells become resistant to a single drug or class of drugs and manifest cross-resistant phenotype to several antineoplastic drugs^4^, that are structurally and functionally unrelated^5^. This phenomenon of acquired drug resistance, known as, multidrug resistance or MDR, is a major challenge faced by cancer therapy till today.

Classical MDR^6^ are characterized by ATP-dependent, unidirectional, membrane-bound drug efflux pumps – the ABC transporter proteins^6,7^. Of 48 ABC genes and 7 subfamilies discovered to date^5^; the most prominent member is Phospho-glycoprotein (also known as Permeability-glycoprotein or P-gp) encoded by the MDR1 (ABCB1) gene^4,6^. P-gp transports a wide range of structurally and functionally divergent chemotherapeutic drugs including vinblastine, doxorubicin, paclitaxel etc.^8^. It effluxes out chemotherapeutic drugs from the cancer cells against steep concentration gradients^9^ resulting in lower intracellular drug concentrations and hence, this truncated drug amount permits cancer cells to survive and proliferate. As such, P-gp overexpression is associated with a negative prognosis in many cancers^10,11^.

P-gp is also reported to induce alterations in other membrane proteins and lipids^12,13^ affecting membrane fluidity and potential, which in turn enhances the effects of P-gp. The cellular localization of P-gp determines the level of resistance to cytotoxic drug accumulation; the plasma membrane expression is associated with higher resistance phenotype^14^ compared to intracellular P-gp. Under normal conditions, P-gp trafficking is highly regulated; sequestering them (P-gp) in narrow membrane domains. Inflammatory diseases lead to a dramatic redistribution of P-gp dependent ATPase activity within the plasma membrane domains^8^ that correlates with it’s ability to translocate drugs across biological membranes^8,15^.

Reversal of MDR by suppression of P-gp could enhance the cellular uptake of anticancer drugs. Strategies implemented to manage MDR include nonionic surfactants or polymers^6,16^, modulators interfering with intracellular ATP production^6,17^, antibodies against MDR proteins, antisense oligonucleotides, ribozymes etc. So far, none of these compounds are effectively shown to overcome drug resistance to the desired level. Moreover, some of the MDR modulators or chemosensitizers^4,6,13^ have been reported to pose systemic toxicity and developing tertiary resistance.

Gene therapeutic technologies provide alternate efficient strategies to circumvent MDR. Small interfering RNAs or siRNAs (21–25 nt long) specifically bind and cleave target mRNAs in a sequence-specific manner, and are powerful tools for gene regulation. In spite of the long half-life of P-gp (5–17 hrs)^18^ transient transfections of siRNA could selectively reduce P-gp in overexpressing Caco-2 cells^18^ and to a greater extent (around 65%) in drug-resistant human breast cancer cells MCF-7/AdrR with an enhanced drug sensitivity^19^. MDR1/P-gp could be down-regulated by shRNAi^20^ and it could be reversed upto 58% in gastric cancer and 89% in pancreatic cancer^21^.

The great potential of siRNAs is thwarted as naked siRNAs are unstable, and are rapidly degraded by nucleases. Also, siRNAs are negatively charged and are challenged by negatively charged cell membrane barrier^22^ and need a protective carrier vehicle for cellular internalization. siRNAs can complex with nanostructures and highly efficient delivery platforms including polymer and lipid-based nanoparticle system^22–24^. Modern nanoparticles also include polymeric micelles^6,7^, liposomes^8,10,25,26^, and lipoprotein-based carriers^9,11^. Nano formulations developed by Chen et al.^27^ delivered both siRNA and Dox to drug-resistant tumors resulting in significant enhancement of drug uptake and anti-cancer effects. The DOPE-PEI nano preparations^28^ encapsulating P-gp siRNA inhibited P-gp expression in resistant breast cancer cell line MCF-7/ADR and enhanced intracellular delivery of Dox.

In order to avoid nonspecific internalization of the nanocomplexes to offset side effects on the non-targeted tissues, these nanoparticles need to be specifically directed to the cancer cells. This cell selectivity can be achieved by aptamer coating^29^ of the nanoparticles. Aptamers are short oligonucleotide sequences (ssDNA or RNA) serving as the targeting ligand that can bind to cell surface antigens. Cancer cells undergo oncogenic mutations leading to the aberrant expression of some cell surface markers. A PCR-based strategy known as Systematic Evolution of Ligands by Exponential enrichment (SELEX) is used to develop aptamers for specific targets^29^. Nanoparticles could be conjugated to the aptamers through an amino or thiol group incorporated at one end of these oligonucleotides during their synthesis. For example, Song et al. has shown an enhanced and targeted delivery of doxorubicin into 4T1 cells by conjugating cell-specific aptamers (SRZ1) with DOTAP-DOPE nanoparticles^30^.

The most useful anticancer drug throughout the world is an anthracyclin called doxorubicin, which intercalates in between double strands of DNA. Dox complexes with and inhibits topoisomerase II after it relaxes supercoiled DNA. The DNA double helix cannot be resealed causing nicks in the DNA preventing replication and leading to apoptosis^10,31^. Other mechanisms of actions of doxorubicin in cancer cells includes the generation of reactive oxygen species that damages cell membranes^25,32^. Dox presents its cytotoxic effects after forming a complex with proteasomes that transport into the nucleus^25,33^.

In our research, we intend to bypass MDR by utilizing the RNAi nanotechnology. Once the drug extruders are silenced, Dox can enter and retain the cells. Earlier, we have developed a liposomal formulation that could transfect liver cancer cells with negligible cytotoxicity and a significant knockdown (85%) capacity of the Hepatitis C Virus (HCV).^34,35^. Also, a systemic administration of combinatorial siRNA nanosomes was noted to considerably reduce HCV replication in a liver tumor-xenotransplant mouse model of HCV without any noticeable liver injury^35^. We have also shown that lipid nanoparticles complexed with high mobility group protein facilitated the delivery of linear and circular DNA into Plasmodium falciparum-infected red blood cells^36^. In the present study, 4T1-R cells, with reduced sensitivity to Dox have been used. The defective Dox uptake is hypothesized to be due to the overexpressed P-gp, which prohibits Dox from cellular internalization. We propose to use polymer substituted lipid nanoparticles with enhanced transfection efficiency and low cytotoxicity to deliver P-gp specific siRNA into the chemoresistant murine breast cancer cells. We have already confirmed the efficacy of this hybrid nanoparticle and functional activity of the encapsulated siRNA in one of our previous works^37^. Silencing of the prominent drug transporter P-gp is expected to increase the Dox accumulation and results in apoptotic induction in resistant cancer cells.

## Materials and Methods

### Materials

The ingredients for nanoparticle preparation were purchased as follows. 1,2-dioleoyl-3-trimethylammonium-propane (DOTAP), Cholesterol and Maleimide-terminated PEG-DSPE (Mal-PEG) from Avanti Polar lipids Inc. (Birmingham, AL, USA), Protamine sulfate salt Grade X, HPLC grade chloroform and Poly (lactide-co-glycolic acid) or PLGA from Sigma Chemical Co. (St. Louis, MO, USA). The cell culture reagents Dulbecco’s modified Eagle’s medium (DMEM) was purchased from Lonza, Fetal bovine serum albumin (BSA) from Mediatech, and penicillin/streptomycin antibiotics were purchased from Gibco, Invitrogen Corp. (Carlsbad, CA, USA). The primary antibody for P-glycoprotein was from Pierce, Thermo Scientific (Waltham, MA) and **β**-actin from Sigma Chemical Co. (St. Louis, MO, USA). The anti-mouse HRP-labeled secondary antibody was purchased from GE Healthcare (Little Chalfont, UK). The MTT reagent was supplied by Sigma Chemical Co. (St. Louis, MO, USA).

### Preparation and characterization of lipid-polymer hybrid nanoparticles

The nanoparticles were prepared by high pressure homogenization (EmulsiFlex-B3) from a mixture of two lipids DOTAP and cholesterol (molar ratio 1:1) with polymer substitution. From the blank liposome, F40 (having an equimolar ratio of DOTAP and cholesterol), eight other formulations were derived - of which four of them is having PLGA (PLGA group) and the remaining four is having PLGA-PEG (PLGA-PEG group)^37^. Considering various particle parameters and cytotoxicity, the best possible combination was F31 belonging to the PLGA group^37^. The procedure of F31 preparation is outlined as follows. This hybrid nanoparticle was prepared 20 mM using 1.8 ml DOTAP (25 mg/ml) and 2.5 ml cholesterol (10 mg/ml) mixed in the molar ratio 1:1, in a round-bottomed flask. The other constituents added to the mixture were 50 **μ**L PLGA (50 mg/ml) and 10 **μ**L Mal-PEG (25 mg/ml). 15 ml HPLC grade chloroform was added and the contents were mixed thoroughly. Then it was dried under nitrogen gas and overnight vacuum. The lipid-polymer film generated was hydrated in DEPC-treated water. The solution was warmed at 50°C and mixed continuously by rotating the flask for 45 min followed by incubation at RT for 2 hrs. Then the solution was homogenized by passing through EmulsiFlex-B3 high pressure homogenizer at 20,000 PSI for 5 cycles. At every cycle, 2.5 ml of the lipid-polymer dispersion was homogenized and the product was collected in another scintillation vial. The final nanoparticle suspension was kept at RT for an hour and then refrigerated and stored at 4°C.

The particle size and surface charge of the nanoparticle F-31 were measured, both before and after siRNA encapsulation using Delsa Nano C Particle Analyzer (Beckman Coulter Inc., Fullerton, CA, USA) at room temperature by dynamic laser light scattering method^37^. The siRNA encapsulation efficiency of this hybrid nanoparticle was also determined by the Ribogreen assay^37^.

### siRNA encapsulation and aptamer-labelling of hybrid nanoparticles

The encapsulation of siRNA into the blank nanoparticles was performed following the same protocol already published earlier^37^. Briefly, freshly prepared protamine sulfate solution (2 **μ**g at 1**μ**g/10 **μl** conc.) in DEPC-treated water was added drop wise to an aqueous solution of siRNA (1 **μ**g at 10 pmol/**μ**l) while vortexing the solution at a moderate speed and kept it at RT for 40 min. The blank nanoparticles were reconstituted into DEPC-treated water and kept at RT for 1 hour. The pre-warmed nanoparticles were briefly sonicated for 2 min in ice and added to the condensed mixture of siRNA-PS (nanoparticle to siRNA ratio 6.8 : 0.66) followed by pipetting 30 times. Then the nanoparticle-siRNA mixture was vortexed slowly 3–4 times to allow thorough mixing and let stand for 15 min at RT. The mixture was sonicated in ice for 2 min to reduce particle size. Finally, the siRNA-encapsulated particles were surface-labeled with aptamer (through binding with Mal-PEG) by incubating the particles with aptamer and mixing through pipetting.

### Cell culture and cell lines

Dox resistant (4T1-R) and dox sensitive (4T1-S) metastatic breast cancer cells were collected from Dr. Srikanta Dash, School of Medicine, Tulane University. All the cells were cultured in high-glucose Dulbecco’s modified Eagle’s medium supplemented with 10% fetal bovine serum, 1% sodium pyruvate, 1% nonessential amino acids, and 1% penicillin- streptomycin and kept at 37°C in a humidified atmosphere with 5% CO_2._

### Cytotoxicity Assay

The cytotoxicity of the 4T1-R and 4T1-S cells was determined in the presence of doxorubicin (Dox) by conversion of the tetrazolium compound to purple formazan by the action of mitochondrial dehydrogenase in actively metabolizing cells. For this purpose, the cells were seeded into 96-well plate at a cell density of 3X10^3^ cells/well in 100 **μ**L media and kept in a 5% CO_2_ incubator at 37°C. The media was changed the next day and added varying concentrations of Dox from 0 to 1.6 **μ**M. The plate was incubated for 48 hrs. Then 20 **μ**L of MTT solution (5 mg/ml in PBS) was added to each well and incubated at 37°C for 2 hrs. After that, the wells were aspirated and formazan crystals were dissolved in 200 **μ**L of dimethylsulphoxide (DMSO) to stop the reaction. Finally, the color intensity was measured at 570 nm using a microplate reader. Each treatment had three replicates and the percentage of cell cytotoxicity was determined to compare to untreated controls.

MTT assay to determine Dox-mediated cytotoxicity was also carried out in 4T1-R cell line after transfecting the cells with siRNA encapsulated nanoparticles with/without aptamer labeling. 3X10^3^ cells/well in 100 **μ**L media were seeded into 96-well plate as above and the next day, nanoparticles encapsulating 100 pmol P-gp siRNA labeled with/without aptamer was added to the cells. After 3 hrs of transfection, the cells were treated with varying concentrations of Dox (0–1.6 **μ**M). Each treatment was carried out in triplicates and the toxicity assay was performed after 48 hours.

### Cell Uptake Study

The intracellular translocation of Dox was monitored in 4T1-R (Dox-resistant) and 4T1-S (Dox-sensitive) cells at three different time points by fluorescence microscopy. In brief, 2X10^5^ cells were seeded into 6-well tissue-culture plates (TCPs) and kept overnight in DMEM supplemented with 10% FBS. The next day, the cells were treated with 1.6 **μ**M Dox and kept at 37°C. After the end of 8, 18 and 36 hours, the cells were washed with PBS, stained with nuclear-dye (Hoechst #33342) and observed under a fluorescent microscope (Olympus IX-71) and photographs were taken at 20X magnification.

For flow cytometric analysis, the cells were treated in a similar fashion and after 18-hour incubation, the cells were scraped using trypsin-EDTA, washed and resuspended in PBS and kept on ice until they were quantified for the percentage of Dox accumulated cells by FACS (BD FACSCalibur).

### Determination of the Expression of P-glycoprotein

#### Immunofluorescence:

The membrane expression of P-gp in 4T1-R and 4T1-S was determined as follows. Approximately 1X10^6^ cells were collected in a 2 ml microcentrifuge tube and washed and incubated for 30 min with the blocking buffer (DMEM + 1%FBS). The primary antibody Anti-P-gp (dilution 1:50) was added in blocking buffer and incubated for 1 hour at 4°C with gentle shaking. Then the cells were washed with blocking buffer and incubated with secondary antibody (dilution 1: 200) for 30 min at 4°C with gentle shaking. The cells were washed with blocking buffer and for fixation resuspended in 2% paraformaldehyde and incubated for 15 min on ice. Finally, the cells were resuspended in blocking buffer (50 **μ**L) and ~30 **μ**L of the cell suspension was put into the glass slide with a cover slip and observed under the microscope.

#### Western Blot:

The cell pellets of 4T1-R and 4T1-S were collected after trypsinization and lysed using appropriate volumes of Million Protein Extraction Buffer (MPer) having protease and phosphatase inhibitors added to it. 30 **μ**g protein from each sample was mixed with SDS loading buffer (Lamelli sample buffer (2X) with 2-Mercaptoethanol. The proteins were separated by Novex 4–20% Tris-Glycine gel and transferred onto a nitrocellulose membrane (Novex, Life Technologies). The membrane was blocked with 5% fat-free milk in Tris-buffered saline with 0.05% Tween-20 (TBS-T) at RT for an hour. The membrane was washed three times with TBS-T and incubated overnight at 4°C with gentle shaking with mouse monoclonal Anti-P-gp antibody (C219; Pierce, Thermo Scientific) at a dilution of 1:1000. The next day, the membrane was washed four times with TBS-T and incubated with an HRP-conjugated mouse secondary antibody at a dilution of 1: 20,000 for 1 hour at RT. The detection of protein expression was carried out using ECL reagent (GE Healthcare) on high-performance chemiluminescence film (Thermo Scientific).

### Transfection of siRNA-encapsulated aptamer-labeled nanoparticles

F31 formulation (encapsulating either P-gp siRNA or GAPDH siRNA) with/without aptamer-labeling was used to transfect 4T1-R cells. In brief, 2X10^5^ cells were seeded into 6-well TCPs and kept overnight. The next day, the cells were replenished with fresh media containing 10% FBS (1 ml) 1 hour before transfection. Protamine-sulphate condensed siRNA was incorporated into the nanoparticle as described in section 2.3. The cells were transfected with 100 **μ**L siRNA encapsulated (100 pmol) aptamer-labeled/non-labeled nanoparticles (nanoparticle: siRNA = 6.8: 0.66) into 900 **μ**L cell culture media supplemented with 10% FBS. After three hours of transfection, 1 ml of growth media was added to the transfected wells. After 24 hours, the cells were washed with PBS, scraped with trypsin-EDTA, pelleted, washed with PBS and kept at −20°C for determination of P-gp expression by western blotting as described in section 2.7.

### Cellular uptake of Doxorubicin after knocking-down P-gp

The intracellular distribution of Dox in 4T1-R was observed by fluorescence microscopy after the targeted delivery of P-gp siRNA loaded F31 nanoparticles with/without aptamer functionalization. The transfection procedure followed has been described in section 2.8. After 3 hours of transfection 1.6 **μ**M Dox was added with 1 ml of fresh media. Some control cells were transfected with the only aptamer in order to check the amount of Dox being delivered by aptamer association. The uptake of Dox in 4T1-R cells after knocking down P-gp by P-gp siRNA was measured by fluorescence microscopy at 24 hr timepoint.

To quantify the intracellular Dox uptake after downregulating P-gp, 4T1-R and MDA-MB-231-R cells were transfected with P-gp siRNA encapsulated nanoparticles with/without aptamer labeling as described in section 2.6 and the percentage of cells having Dox was determined by FACS analysis. The concentration of Dox used was 1.6 **μ**M and added after 3 hours of transfection. After 18 hours of Dox treatment, the cells were trypsinized, washed with PBS and then resuspended in PBS and kept on ice until they were subjected to flow analysis (BD FACSCalibur). Each treatment was done in triplicates.

## Results

The lipid-polymer hybrid formulation used for targeted delivery, F-31, has been selected from a group of nine formulations previously developed and characterized in our lab^37^. Starting from the lipid formulation F40, the lipid-polymer derivatives with PLGA and PLGA-PEG were synthesized by substitution of lipids by a polymer. These nano formulations have been characterized for particle size, surface charge, siRNA encapsulation efficiency and cytotoxicity^37^. F-31 was chosen for its smaller particle size post siRNA encapsulation and aptamer labeling, which leads to improved delivery efficiency. Also, its siRNA encapsulation efficiency was higher and cytotoxicity was lower as compared to others.

### Cytotoxic effects of Doxorubicin in drug-resistant and drug-sensitive cell lines

**Figure 1.**
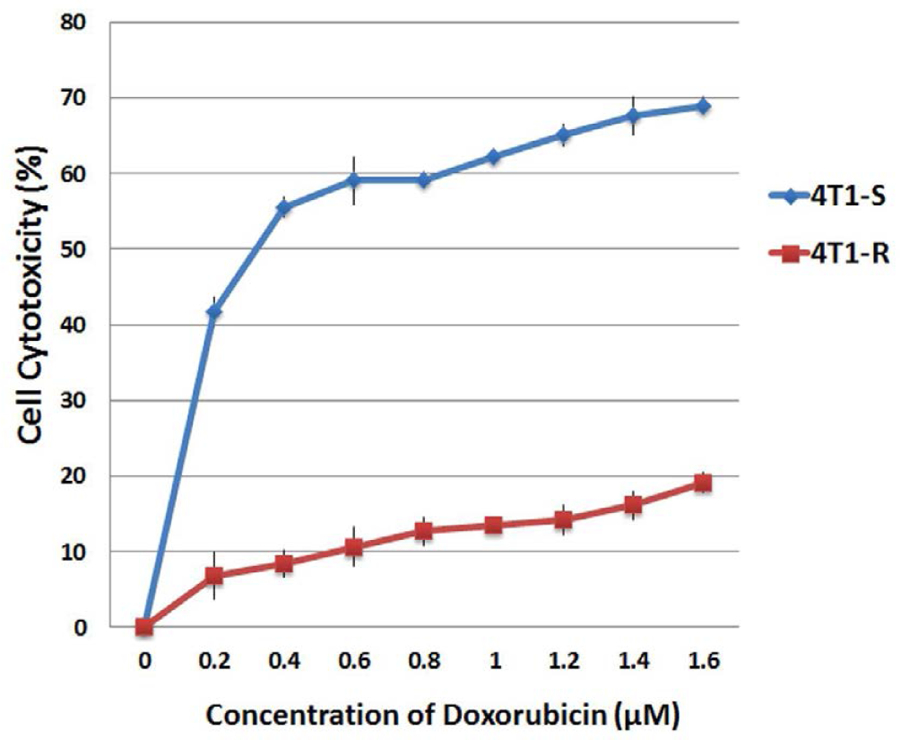
Comparing cytotoxicity of doxorubicin on Dox-resistant 4T1-R and Dox-sensitive 4T1-S breast cancer cells. The cells were treated with increasing concentration of doxorubicin for 48 hrs and the cell cytotoxicity was measured by MTT assay following the manufacturer’s protocol. The results represent mean ± standard deviation (n = 3).

The doxorubicin-induced cytotoxicity was evaluated in murine breast cancer cell lines 4T1-R (Dox-resistant) and 4T1-S (Dox-sensitive) by MTT assay after 48 hours of treatment. The 4T1-R cells treated with increasing concentration of Dox (0–1.6 **μ**M) showed very feeble evidence of cytotoxicity (Fig. 1) ranging from 8% with 0.2 **μ**M to 20% with 1.6 **μ**M. In sharp contrast, Dox-induced a significant dose-dependent cytotoxicity in 4T1-S, which is sensitive to Dox. The percent cytotoxicity in 4T1-S ranged from 41% with 0.2 **μ**M to 69% with 1.6 **μ**M respectively. The half-maximal inhibitory concentration (IC_50_) of Dox in 4T1-S was reached at 0.4 **μ**M.

### Differential translocation of Doxorubicin in 4T1 resistant and sensitive cell lines

**Figure 2.**
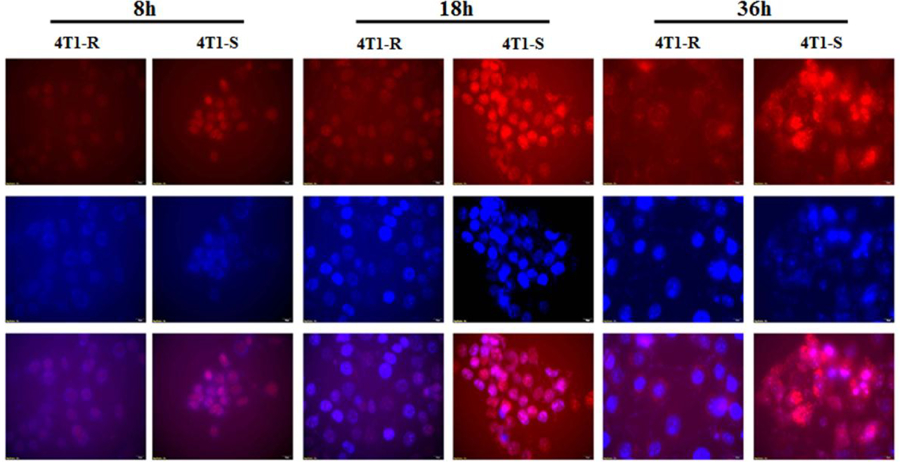
Comparing the intracellular translocation of doxorubicin into Dox-resistant 4T1-R and Dox-sensitive 4T1-S cells at different time points. The cells were treated with 1.6 μM doxorubicin and at the end of 8, 18 and 36 hrs, the translocation of doxorubicin into the cells was observed under a fluorescent microscope at 20X magnification.

To understand the doxorubicin resistance from a mechanistic point of view, a kinetic study was launched to compare the cellular uptake of doxorubicin between 4T1-R and 4T1-S cells. The cells seeded in TCPs as described in section 2.6, were treated with 1.6 **μ**M Dox, and at the end of 8, 18 and 36 hrs, the intracellular trafficking of Dox was monitored by fluorescence microscopy (Fig. 2). The top panel shows images of intracellular Dox fluorescence. After 8 hrs timepoint, nuclear translocation of Dox was observed in 4T1-S and a very minute distribution of Dox was noticed in 4T1-R. The contrast between 4T1-R and 4T1-S cells was sharp at 18 hrs timepoint, with significantly high fluorescence of Dox in 4T1-S cells but only a feeble fluorescence of Dox in 4T1-R. Though the accumulation of Dox in 4T1-S is higher at 36 hrs compared to 8 and 18 hrs, the image is a bit diffused due to Dox-induced high cytotoxicity and consequent cell death. Very low intracellular accumulation of Dox is observed in 4T1-R even after 36 hrs in marked contrast to 4T1-S. Hoechst 33342 has been used to stain the nuclei and photographed in the middle panel. The lower panel in Fig. 2 shows a combination of Dox and nuclear stain.

**Figure 3.**
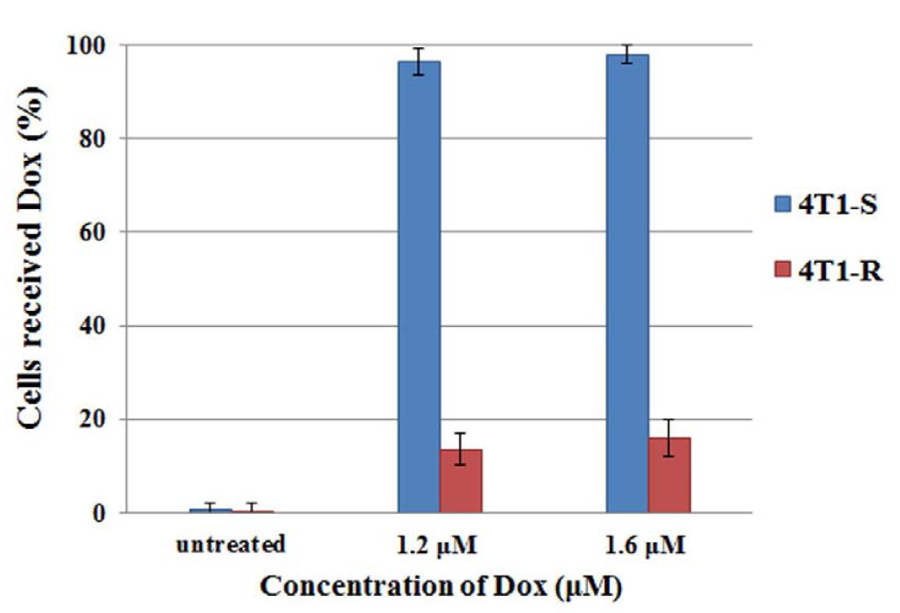
Quantitative comparison of doxorubicin update by Dox-resistant 4T1-R and Dox-sensitive 4T1-S cells by FACS analysis. After treating with 1.6 μM doxorubicin for 18 hrs, the percentage of cells with Dox accumulation was quantified by FACS analysis

A quantitative analysis of intracellular Dox fluorescence was carried out by FACS analysis in order to further substantiate the results observed by fluorescence microscopy in 4T1-R and 4T1-S cells. The FACS analysis was carried out after18 hrs of Dox treatment (Fig. 3) as a distinct difference in intracellular distribution of Dox between the resistant and sensitive cell lines were observed at this timepoint by fluorescence microscopy. The FACS analysis data (Fig. 3) corroborates with the fluorescence microscopic studies. Dox accumulation was observed in almost ≈100% of the 4T1-S cells while only ≈20% of the 4T1-R cells seemed to take up the drug.

From the results of fluorescence microscopic studies and flow cytometry, it is evident that Dox is able to cross the cell membrane and efficiently localizes in the nucleus of 4T1-S cells thereby attributing to its high-level cytotoxicity as observed by MTT assay (Fig. 1). In the resistant 4T1-R cell line, it is probable that Dox is either unable to cross the cell membrane in a sufficient number or is hindered from reaching the nucleus and is concentrated exclusively in the cytoplasm. This suggestion stems from the fact that P-gp is localized both at the cellular and nuclear membranes^38^.

### Expression of Drug Efflux Pump

The expression of the drug efflux proteins like P-gp could be the hidden factor behind this impaired Dox uptake in 4T1-R cells. After treatment with Dox solution, cellular efflux was faster in P-gp overexpressing cell lines^39^. As such the expression of P-gp at the protein level was assessed by immunofluorescence and western blot. A high level of membrane expression of P-gp was observed in Dox-resistant murine 4T1-R cells as well as Dox-resistant human MDA MB-231-R breast cancer cells (Fig. 4A). Conversely, comparatively a low expression of P-gp was observed in Dox-sensitive 4T1-S cell line as well as Dox-resistant human MDA MB-231-S cell line by western blotting (Fig. 4A).

The comparative higher expression of P-gp in 4T1-R over 4T1-S and MDA MB-231-R over MDA MB-231-S was also confirmed by fluorescence microscopy (Fig. 4B) which attributes the higher resistance of Dox accumulation into the drug-resistant 4T1-R and MDA-MB-231-R cells. Inhibiting the expression of P-gp is expected to increase the cellular translocation of Dox and consequent cytotoxicity.

### Downregulation of the expression of P-gp in resistant cell lines by delivery of siRNA using aptamer-labeled nanoparticles

**Figure 5.**
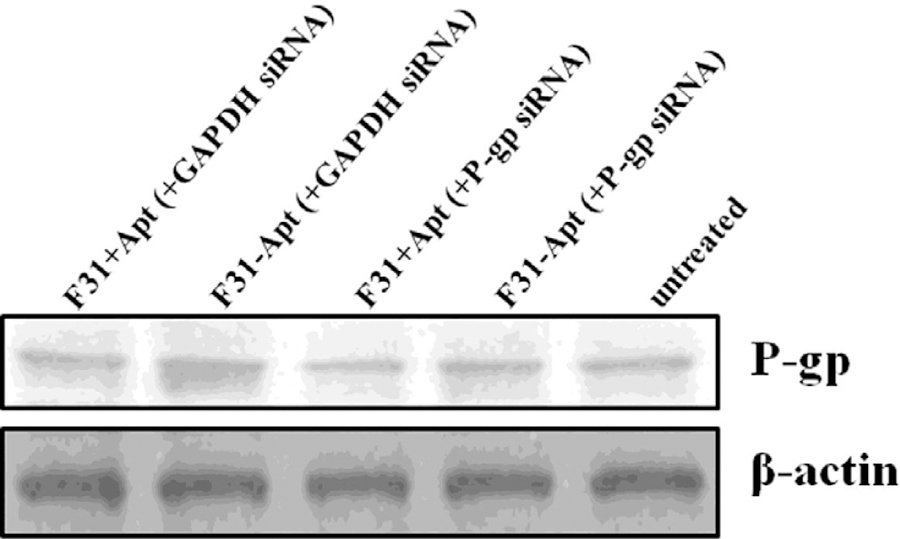
Measuring the knockdown of P-gp in 4T1-R cells by the nanoparticles (encapsulating either P-gp siRNA or GAPDH siRNA) with/without aptamer labeling. The cells were transfected with 100 pmol (P-gp or GAPDH) siRNA-encapsulated aptamer-labeled/non-labeled nanoparticles for 24 hrs followed by Western blotting to measure the expression of P-gp.

The lipid-polymer hybrid nanoparticles F31, after encapsulating P-gp siRNA and surface-labeled with Apt-A6 were used to transfect Dox-resistant breast cancer cells 4T1-R. The siRNA chosen to knockdown P-gp has passed a high throughput screening procedure and has proven efficacy of silencing P-gp in MDR breast cancer cells^40^. The delivery efficiency of these nanoparticles and the functional activity of siRNA in three different cell lines have been assessed in our earlier studies^37^. To confirm that the silencing effect is brought about by only target-specific siRNA, an unrelated siRNA (GAPDH siRNA) transfection has also been compared (Fig. 5).

It is evident from the results of western blot that the siRNA delivered by aptamer-labeled lipid-polymer hybrid nanoparticles has been successful in partial silencing of P-gp expression in 4T1-R cells (Fig. 5). The specificity of the P-gp siRNA is also confirmed as GAPDH siRNA delivered in a similar manner has proved to be ineffective to knockdown P-gp.

### Improved uptake of doxorubicin in Dox-resistant cells following P-gp knockdown

The Dox-resistant murine carcinoma cells, 4T1-R originally showed impediment in Dox uptake (Fig. 2, Fig. 3) as compared to the significantly higher uptake of Dox by Dox-sensitive 4T1-S cells. This resistance of Dox accumulation stems from the overexpression of P-gp (Fig. 4A & 4B) on the plasma membrane of 4T1-R cells. Partial silencing of P-gp (Fig. 5) was observed in the dox-resistant cell lines by western blotting after the targeted delivery of P-gp siRNA using aptamer-labeled nanoparticles.

**Figure 6.**
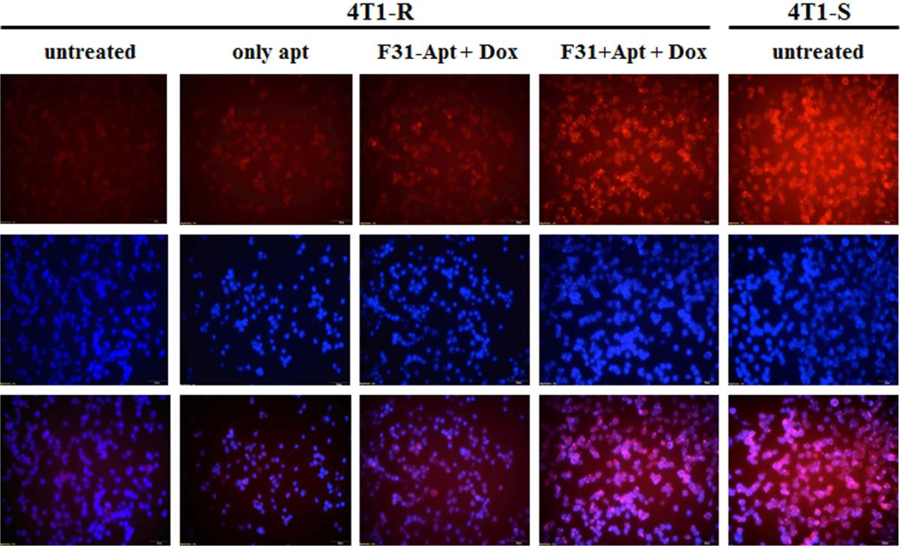
Comparing the intracellular translocation of doxorubicin after knocking-down P-gp into the Dox-resistant 4T1-R breast cancer cells. After knocking-down P-gp by P-gp siRNA-encapsulated nanoparticles with/without aptamer labeling, the cells were treated with doxorubicin and the uptake of Dox was measured by fluorescence microscopy after 24 hrs at 10x magnification.

The cellular trafficking of Dox was monitored in 4T1-R cells by fluorescence microscopy, after knocking down P-gp with/without aptamer-labeled nanoparticles (Fig. 6). Aptamer labeling on the surface of the nanoparticles enhances the delivery efficiency^37^ and leads to better knockdown of the target protein (Fig. 5). It is obvious from fluorescence microscopic studies that there is a marked increase in Dox uptake after the downregulation of P-gp (by non-aptamer labeled siRNA-encapsulated nanoparticles) compared to untreated (F31-Apt + Dox vs. Untreated) (Fig. 6). Dox translocation in 4T1-R cells is enhanced even more when P-gp siRNA is delivered by aptamer-labeled nanocarriers (F31+Apt + Dox vs. F31-Apt+Dox). The overlapping accumulation of Dox (3^rd^ row, Fig. 6) with the nuclear staining (2^nd^ row, Fig. 6) seen in those images confirms the nuclear localization of Dox into the Dox-resistant 4T1-R breast cancer cells.

**Figure 7.**
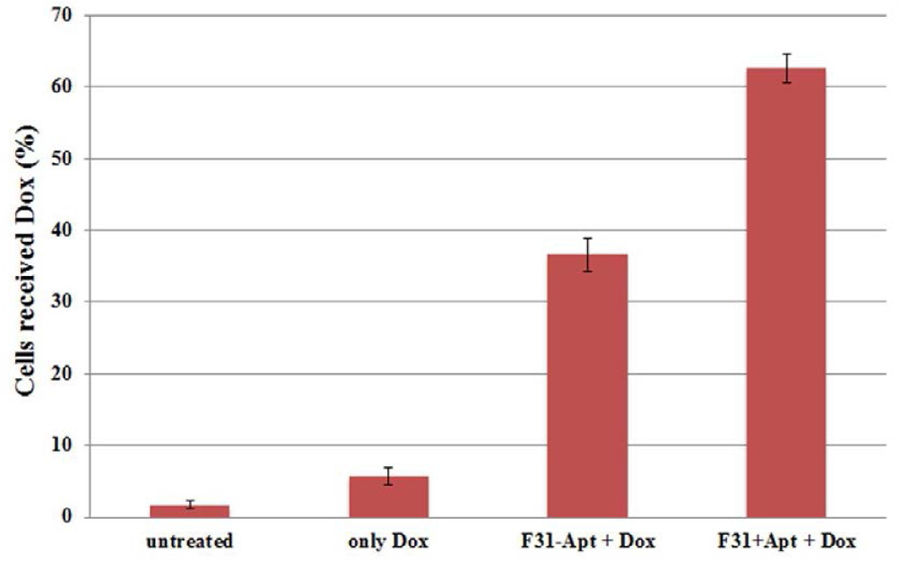
Quantitative comparison of doxorubicin update by Dox-resistant 4T1-R cells after knocking-down P-gp by P-gp siRNA-encapsulated nanoparticles (with/without aptamer labeling) by FACS analysis. The cells were transfected with P-gp siRNA-encapsulated nanoparticles with/without aptamer labeling followed by Dox treatment for 24 hrs. After that, the percentage of cells uptaking Dox was quantified by FACS analysis.

The quantitative evaluation of intracellular doxorubicin fluorescence after partial silencing of P-gp was also measured by flow cytometry (Fig. 7). The results indicate that knocking down P-gp by non-targeted P-gp siRNA-encapsulated hybrid nanoparticles (F31-Apt + Dox) has successfully increased the Dox uptake from ≈8% (only Dox) to ≈36% (F31-Apt + Dox) in those Dox-resistant 4T1-R cells. Again, a further improvement is noticed when ≈62% of the cells registered positive for Dox fluorescence when the 4T1-R cells were transfected with aptamer-labeled siRNA encapsulated nanocarriers (F31+Apt + Dox).

### Status of P-gp expression after Dox treatment

**Figure 8.**
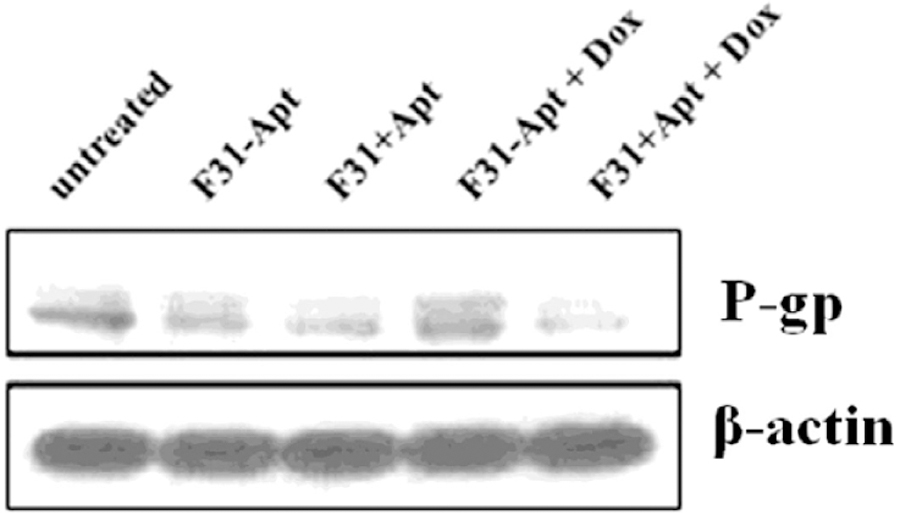
Comparing the expression of P-gp in Dox-resistant 4T1-R breast cancer cells after knocking down P-gp by nanoparticles with/without aptamer-labeling followed by Dox treatment. The 4T1-R breast cancer cells were transfected with aptamer/non-aptamer labeled P-gp siRNA encapsulated nanoparticles for 24 hours followed by another 3 hours with/without Dox treatment. Then the cells were scraped by using trypsin-EDTA, washed, pelleted and the expression of P-gp was measured by Western blot analysis.

To find out whether the aptamer labeling of the nanoparticles can enhance the silencing of P-gp and sustain the knockdown activity even at Dox exposure, Dox-resistant 4T1-R breast cancer cells were transfected with aptamer/non-aptamer labeled P-gp siRNA encapsulated nanoparticles for 24 hours followed by another 3 hours with/without Dox treatment. The Western blot results shown in Fig. 8 indicate a distinguishable decrease in P-gp expression in 4T1-R cells when the cells were transfected with aptamer-labeled siRNA-encapsulated nanoparticles (lane 3; F31+Apt) compare to untreated (lane 1) and non-aptamer labeled siRNA-encapsulated nanoparticles (lane 2; F31-Apt). On the other hand, when the cells were exposed to Dox for 3 hours following transfection with siRNA encapsulated nanocarriers for 24 h, a significant difference in P-gp expression was observed between aptamer-labeled and non-aptamer-labeled nanoparticles transfection (Fig. 8). The low expression profile of P-gp was maintained in aptamer-labeled siRNA-encapsulated nanoparticle transfection even after Dox exposure (lane 5; F31+Apt+Dox), whereas, the non-aptamer labeled siRNA-encapsulated nanoparticle transfection could not sustain the P-gp silencing, as presumably, the Dox can enhance the expression of P-gp in those Drug-resistant 4T1-R breast cancer cells (lane 4; F31-Apt+Dox in Fig. 8).

### Enhanced cytotoxicity of Doxorubicin in resistant cell lines after partial silencing of P-gp by targeted delivery of P-gp siRNA using aptamer/non-aptamer labeled nanoparticles

The membrane expression of P-gp in Dox-resistant cell lines 4T1-R has been attributed to be a major contributing factor behind impediment in Dox uptake. Suppression of P-gp expression (Fig. 5) using siRNA delivered by aptamer-labeled hybrid nanocarriers has been found to facilitate intracellular translocation of Dox (Fig. 6 & Fig. 7) in Dox-resistant 4T1-R cells. Sensitization of the resistant cells to Dox is expected to improve the Dox-induced cytotoxicity in the Dox-resistant 4T1-R cells.

**Figure 9.**
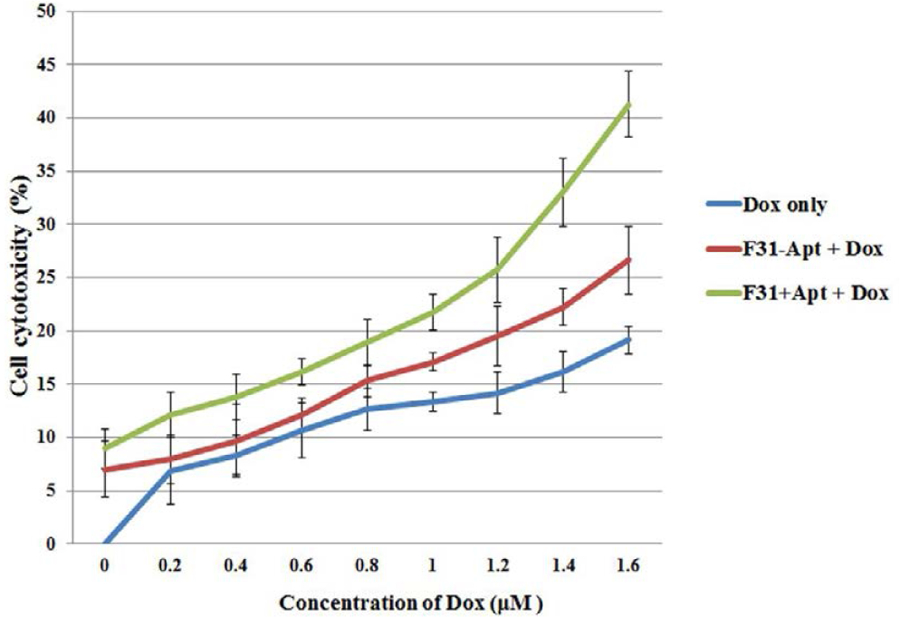
Comparing the cytotoxicity of Dox on the Dox-resistant 4T1-R breast cancer cells after silencing P-gp by P-gp siRNA-encapsulated nanoparticles with/without aptamer labeling. The 4T1-R cells were treated with nanoparticles encapsulating P-gp siRNA with/without aptamer functionalization and were subjected to varying doses of Dox treatment (0–1.6 **μ**M) after 3 hrs of transfection with siRNA. After 24 hrs, the cell cytotoxicity was measured by MTT assay following the manufacturer’s protocol.

The 4T1-R cells were treated with nanoparticles encapsulating siRNA with/without aptamer functionalization and were subjected to varying doses of Dox treatment (0–1.6 **μ**M) after 3 hrs of transfection with siRNA. Dose-dependent cytotoxicity of Dox in 4T1-R cells has been compared between transfections with and without aptamer-labeled P-gp siRNA encapsulated nanoparticles (Fig. 9). A sharp increase in Dox-induced cytotoxicity (statistically significant) is noticed when the 4T1-R cells were transfected with aptamer-labeled nanoparticles compared to the particles with no aptamer-labeling. Side by side, the cells treated with Dox only showed lower cytotoxicity compared to those treated with Dox following the transfection with siRNA encapsulated nanoparticles with/without aptamer-labeling.

## Discussion

A major obstacle in the chemotherapeutic treatment of cancer is the gradual development of multi-drug resistance (MDR) due to which the treatment loses its efficacy leading to poor prognosis^18,41^ and clinical outcomes. There are several types of drug resistance, the most well known are the efflux pump type resistance, repair of the damaged DNA and apoptotic inhibition. The present study is concerned with the MDR events triggered by overexpression of P-gp^18,42^; a transmembrane efflux transporter, which significantly attenuates intracellular drug accumulation leading to suboptimal therapeutic responses. P-gp expression has been noticed in over 50% of cancers with MDR phenotype^43^. Newly diagnosed breast carcinoma shows a 0–29% incidence of P-gp expression but this can increase to 71% at relapse. Around 90% of the anticancer drugs are P-gp substrates and of them, doxorubicin^43^ is most commonly used in chemotherapy of most cancers. The level of P-gp expression in breast carcinoma directly correlates to levels of tumor resistance to P-gp substrate like Dox^44^.

The present day cancer therapy counts on reversing MDR events in its attempt to overcome drug resistance. Increasing the drug dose will not help to circumvent this P-gp mediated drug efflux. Rather, an appropriate therapeutic ratio of cancer chemotherapy could be derived through alternative approaches that may improve uptake and prolong retention of cytotoxic drugs in resistant cancer cells. The RNA interference is a strategic approach for transient silencing of target gene expression at the post-transcriptional level and for reducing pump mediated drug efflux. This sequence-specific gene silencing offers a relatively safe method for the downregulation of P-gp without side effects.

siRNAs are negatively charged and are challenged during crossing the cell membrane. Their short half-life coupled with poor cellular uptake limits their therapeutic efficacy. Nano formulations composed of cationic lipids and polymers can complex with anionic siRNAs via ionic interactions. The nucleic acids encapsulated in a protective shell could be delivered with ease to mammalian cells thereby increasing transfection efficiency. The siRNAs encapsulated within nanoparticles could escape RNase degradation and renal clearance and undergo controlled-release with an increased half-life in the bloodstream^45,46^. The addition of FDA approved, biocompatible polymer, PLGA greatly increases the physiological stability and biological safety of the nanocarriers over cationic lipids^47^. PLGA that we have used to make the formulations is composed of monomers lactic acid and glycolic acid combined at the ratio of 65:35. The interparticle fusion leading to the self-decomposition of the liposomes is supposed to be minimized due to polymer substitution, which stabilizes it. The cytotoxicity profile of hybrid lipid-polymer nanoparticles is also improved over that of liposomes. This enhanced interfacial stabilization due to PLGA substitution decreases the particle size and improves siRNA encapsulation efficiency^37^. Nanoparticles are advantageous not only for their ability to assemble siRNAs within the stacks of lipid bilayers but also for their large surface areas that could be functionalized to associate with aptamers. Aptamers selectively recognize and bind to certain surface markers overexpressed in specific cells. Her-2 overexpression is observed in 25% of invasive breast cancer and is, therefore, one of the main targets for nanoparticle delivery. For selectively targeting breast cancer cells, our nanocarriers have been labeled with aptamer A6, which recognizes and binds to molecular signatures (like Her-2) overexpressed on breast cancer cells. Aptamers with the help of their terminal amino (–NH_2_) groups associate with Mal-PEG already incorporated into the nanoparticles. These nanoparticles are internalized through cell-mediated endocytosis^34^.

From the basic liposome formulation F40, the new lipid-polymer hybrid nanoparticles were formulated replacing lipids by varying amount of PLGA or PLGA-PEG^37^. F31 and F21 were chosen as the most effective formulations from respective PLGA and PLGA-PEG groups. F31, after siRNA encapsulation and aptamer labeling, was found to be smaller in particle size and had better siRNA retention capacity than F21. Cellular toxicity studies using different siRNA : particle : aptamer ratios showed better results for F31 than corresponding F21 preparations^37^. As such, we have used F31 formulations for siRNA transfections in the current study. This lipid-PLGA hybrid formulation, F31 was able to efficiently transfect 4T1-R cells as confirmed in our previous study. Therefore, these nanoparticles were assembled with P-gp siRNA to silence P-gp. The siRNA used for knocking down P-gp expression was actually selected by Meng et al.^40^ from a panel of siRNAs by high throughput screening of their efficiency of silencing P-gp in a multi-drug resistant breast cancer cell line that maintained stable P-gp expression. In one of our previous publications^37^, we have shown that aptamer functionalization of the nanoparticle surface has significantly increased the delivery of siRNA into the breast cancer cell. Therefore, we have used F31 preparations labeled with aptamer A6 for targeted delivery into breast cancer cells. These nanoparticles are effective even under high serum conditions (10% FBS) and could guard the entrapped siRNA against serum nucleases^37^.

In the present study, we used a highly metastatic 4T1 murine mammary carcinoma cell line that closely resembles that of advanced human breast cancer stage IV. The 4T1-R cells were actually isolated from mouse tumors resistant to Dox and cultured in growth medium supplemented with Dox to make them truly Dox-resistant^48^. The 4T1-R cells do not undergo G2/M phase growth arrest. Side by side we used 4T1-S cells, for comparison, which were highly Dox-sensitive and undergo G2/M phase growth arrest. The differential intracellular translocation of Dox in the resistant and sensitive cell lines resulted in different cytotoxic effects. 4T1-R cells showed resistance to nuclear entry of Dox and subsequent DNA damage even for prolonged Dox exposure. This impaired nuclear translocation of Dox in 4T1-R cells has also been observed by other investigators^48,49^.

High expression of P-gp is attributed to the defective nuclear translocation of Dox in the resistant cell line^48,50^, which is one of the established mechanisms of chemoresistance. The Dox-resistant P-gp needs to be silenced, before drug treatment. We have initially transfected 4T1-R cells using varying concentrations of siRNA (25, 50, 75, 100 pmol), varying siRNA: nanoparticle ratios for different time periods (data not shown). Noticeable knockdown was achieved at a nanoparticle: siRNA ratio of 6.8: 0.66, siRNA concentration of 100 pmol and a time period of 24 hrs incubation.

In the synergistic treatment approach of siRNA and anticancer drug Dox, it is crucial that both siRNA and Dox are simultaneously delivered to the drug-resistant cells and that siRNA can freely downregulate P-gp, before P-gp’s reactivation in presence of Dox. Therefore, we have transfected the cells with the P-gp siRNA loaded nanocarriers earlier so that the P-gp expression could be inhibited before Dox treatment and the drug should be retained from getting pumped out. A time-lapse of 3 hours has been maintained between P-gp siRNA transfection and drug treatment. Again, the release kinetics of siRNA from the nanocarriers is complex and time-consuming while Dox enters the cell by passive diffusion and it takes about 15 min^30^ for Dox entry. Again, the intracellular siRNA stability (half-life of siRNA) is not a contributing factor while cell division of rapidly dividing cells (like 4T1-R) is a limiting factor in gene silencing duration^51^. Since, the downregulation of P-gp is transient, the drug sensitizing effect will last for a window of time within which Dox needs to be taken up by the otherwise resistant cancer cell. Precise control of timings is very important and the time gap maintained in our study between transfection and drug treatment seemed to match. Moreover, Dox and siRNA simultaneously remain in the cells for sufficient time giving rise to co-operative effects on cancer cells. As a result, efficient reduction of P-gp expression and cell apoptotic induction in the MDR cell line is achieved leading to the restoration of chemosensitivity.

In the present study, Dox is not conjugated with the aptamer, but is added separately after 3 hrs of transfection with siRNA encapsulated nanoparticles and is expected to be taken up by cells within 15 min via passive diffusion (Fig. 6)^30^. There is a possibility that Dox could be intercalating into the folded structure of the aptamer^29^ and entering the cells by binding with free aptamer present in the media after transfection. We have used an aptamer only control so as to know the percent of Dox entered by Dox-aptamer association (Fig. 6). A small increase in Dox fluorescence is observed with the only aptamer, but is much lower than what is observed after P-gp knockdown using targeted nanoparticles. Therefore downregulation of P-gp using siRNA encapsulated nanocarriers sensitizes 4T1-R cells to Dox. The drug was only observed to intercalate in the nucleus in the resistant cell line after P-gp knockdown (Fig. 6).

The aptamer-functionalized lipid-polymer hybrid nanoparticle with entrapped P-gp specific siRNA could successfully downregulate P-gp expression. Detection of P-gp has been carried out by western blot and the major band of the P-gp was detected around 55 k Da. This was slightly intriguing as P-gp is a 170 kDa protein and is usually detected around 140 kDa. Several investigators have shown that due to proteolytic digestion with trypsin, P-gp gets cleaved into smaller fragments of mol. Wt. of 95 and 55 KDa^52^, 70, 55 and 40 kDa^53^; 95 and 54–56 kDa^54^. With trypsinization, the intensity of the higher band (~140 kDa) decreases and the lower band increases. Enhanced trafficking of Dox is visualized after the downregulation of P-gp. The delivery of siRNA and subsequent silencing of P-gp is higher with the aptamer functionalization of the nanoparticles than with those devoid of it.

The knockdown in gene expression should reach a certain threshold before the therapeutic effect is realized. In the system being considered, P-gp is a protein with a long half-life (5–17 hours), and maybe multiple doses of siRNA could be necessary to achieve a stable knockdown for in vivo experiments. It is noticed that even after the downregulation of P-gp, though there is an enhancement in Dox fluorescence in 4T1-R cells, it is still lower than what is observed in 4T1-S cells (Fig. 6). This could be due to the presence of other drug-transporters like MRP-1 on the membrane of 4T1-R cells. P-gp substrate Dox could be a substrate to other efflux pumps as well. Therefore, even after downregulation of P-gp, efflux by other pumps may still occur. Also, with our delivery system we could achieve only a partial silencing of P-gp. P-gp is encoded by two different genes in rodents, mdr1a and mdr1b, with partly overlapping substrate specificity and efflux efficiency. In this study, we have focused on delivering siRNA targeted to mdr1a, so the other gene mdr1b could still have been active. Again, as 4T1-R has been exposed to Dox for over a long period of time, additional mechanisms of drug resistance may be active including inhibition of apoptosis, repair of the damaged DNA and alteration of the cell cycle checkpoints.

## Conclusion

The present study suggests a novel aptamer-labeled hybrid lipid-polymer formulation for overcoming the drug resistance of P-gp overexpressing tumor cells. The feasibility and effectiveness of this nanocarrier system have been confirmed by P-gp silencing by siRNA delivery. Administration of siRNA nanocomplexes labeled with aptamer could inhibit P-gp expression more efficiently than only nanocomplexes (without aptamer labeling) containing siRNA. The silencing of P-gp by aptamer-labeled siRNA-encapsulated nanoparticles compare to non-aptamer-labeled siRNA encapsulated nanoparticles leads to effective inhibition of its efflux activity with an enhancement of intracellular Dox accumulation and apoptotic induction in resistant murine breast cancer cell. These results suggest that the use of P-gp siRNA loaded nanocomplexes labeled with aptamer may be a promising gene delivery strategy to reverse MDR and improve the effectiveness of chemotherapy. However, the therapeutic efficacy of chemotherapy is limited due to different forms of MDR, and downregulation of one gene (P-gp) may intensify the expression of other drug transporters. Our future goal is to develop aptamer-labeled hybrid nano formulations, which could encapsulate multiple siRNAs targeted to different ABC transporters like MRP-1 and BCRP in addition to P-gp so as to push for a co-operative enhanced effect with further improvement in drug uptake.

## Figures and Tables

**Figure 4A.**
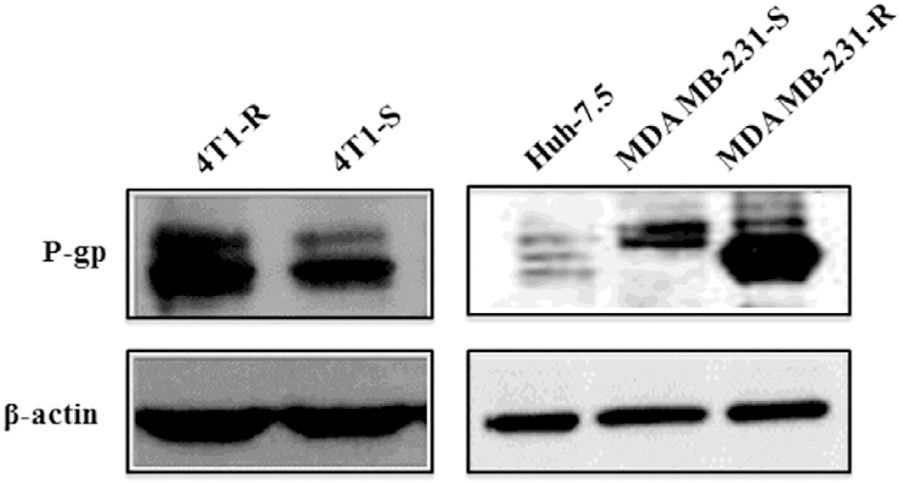
Comparing the expression of P-gp in different mouse (i.e. 4T1-R and 4T1-S) and human breast cancer cells (i.e. MDA MB-231-R and MDA MB-231-S) as well as Huh-7.5 human liver cancer cells by Western blot analysis. After 24 hrs of cell culture, the cells were scraped, lysed, and equal amount of proteins was loaded to measure the expression of P-gp (1: 1000) with β-actin (1:20,000) running as the loading control.

**Figure 4B.**
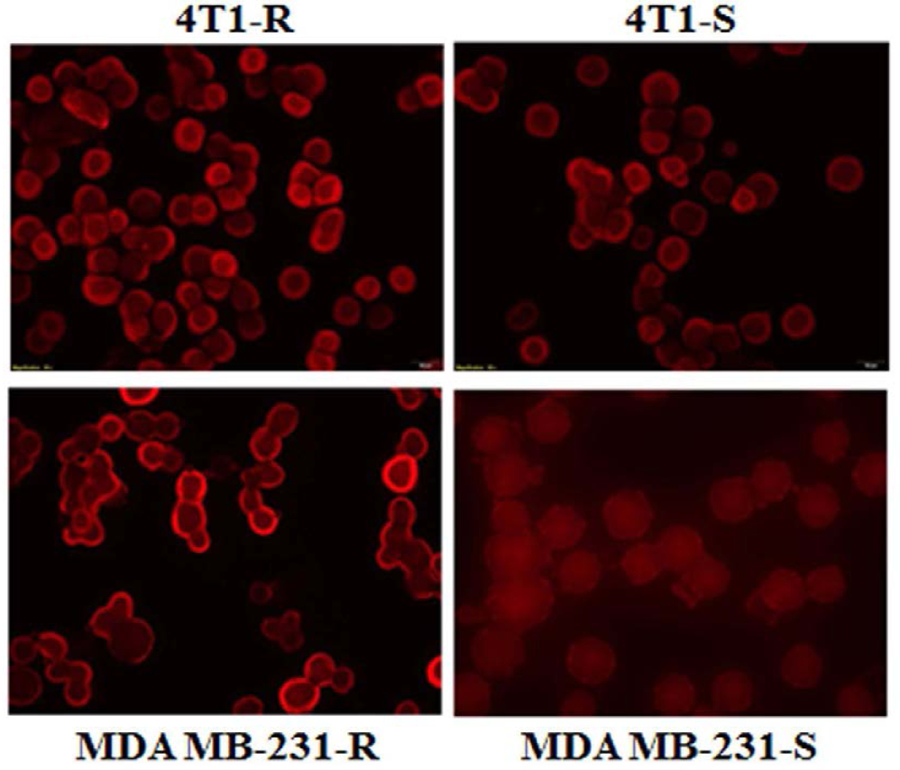
Comparing the expression of P-gp on Dox-resistant 4T1-R (mouse) and MDA MB-231-R (human) cells vs. Dox-sensitive 4T1-S (mouse) and MDA MB-231-S (human) cells by fluorescence microscopy at 20x magnification.
